# Therapeutic writing in a programme for binge eating disorder—A tool to come closer to clarifying feelings

**DOI:** 10.1111/scs.13095

**Published:** 2022-06-23

**Authors:** Eileen Oftedal, Venke Ueland, Kristine Rørtveit

**Affiliations:** ^1^ Faculty of Health Sciences Department of Caring and Ethics Life Phenomena and Caring Research Group University of Stavanger, UIS Stavanger Norway; ^2^ Stavanger Community Mental Health Centre Stavanger University Hospital Stavanger Norway; ^3^ Research Department Nursing and Health Care Research Group Stavanger University Hospital Stavanger Norway

**Keywords:** affect consciousness, binge eating disorder, cognitive behavioural therapy, group therapy, qualitative study, therapeutic writing

## Abstract

**Introduction:**

Therapeutic writing involving affect consciousness (AC) can be used to put difficult topics into words. In this study, we investigated how patients with binge eating disorder (BED) experienced therapeutic writing and AC in the context of cognitive behavioural therapy. The elements were included in an existing cognitive behavioural therapy group programme and the participants' experiences investigated qualitatively.

**Aim:**

To investigate therapeutic writing as experienced by patients in the context of a BED group programme focusing on AC.

**Method:**

A phenomenological, hermeneutic design with semi‐structured interviews was employed. Eight participants were recruited after completing the BED programme at a Community Mental Health Centre on the West Coast of Norway.

**Findings:**

Four sub‐themes emerged: *Struggling to achieve a flow in the writing process, Deeper understanding of eating patterns through writing, Moving specific feelings towards the surface by writing* and *Greater insight into oneself as a human being by shared writing.* Based on the sub‐themes, one main theme was developed: *Therapeutic writing in a binge eating disorder programme means focusing on oneself as a human being by becoming closer to one's feelings.*

**Conclusion:**

Therapeutic writing offered in treatment for BED involves individual movements at different levels, including processes of awareness of feelings, cognitions and oneself as a human being.

## INTRODUCTION

In the present study, experiences of therapeutic writing involving affect consciousness (AC) were examined in the context of cognitive behavioural therapy (CBT) for patients with binge eating disorder (BED) [1].

BED is an eating disorder characterised by uncontrolled binge eating episodes and lack of control during eating [2, 3]. Overeating is the main feature of BED, where uncontrolled overeating episodes and the absence of weight control methods such as purging or overtraining are central [2, 3]. According to the International Classification of Diseases, overeating episodes occurring once a week indicate a BED diagnosis [4]. There is strong empirical evidence for the association between negative effects and BED [5, 6, 7, 8, 9, 10]. The process of overeating is self‐reinforcing [2, 3] and can be an attempt to downregulate emotional stress in the absence of healthy and effective emotion–regulation strategies [11].

### Study background

The programme has an eclectic approach as we selected doctrines from various models: therapeutic writing, CBT and AC. We included therapeutic writing as a means to integrate affects in CBT offered to patients with BED [1]. Therapeutic writing has been provided in other clinical settings such as groups working with personal grief [12‐14] or long‐term pain [13, 14, 15]. Therapeutic writing is a creative act comprising different personal writing activities that provide insight or perspective on life [16], help one to deal with emotionally upsetting events [17] and is personal, private and free from criticism [18].

AC means being able to consciously perceive, bear, reflect upon, enunciate and express experienced affective states [19]. The integrating aspects of AC have been operationalised for different affect categories as degrees of awareness, tolerance, emotional and conceptual expression. The AC model is a psychotherapy model with special focus on affect integration [20, 21, 22]. Feelings not integrated into mental representations lead to unclear and impersonal communication with one self and others [22]. Patients seem more involved in the dialogue and focus better when the therapy offers ongoing exploration of affect experiences. By repetition, patients learn the benefit of affect focus and eventually relate to their feelings in a more appropriate way [19].

CBT is a problem‐solving intervention that aims to improve mental health by challenging thoughts, beliefs, attitudes and behaviour. It has been documented as best practice for BED [23, 24]. CBT provides a better long‐term outcome, greater reduction of overeating symptoms and better quality of life [25, 26, 27, 28]. CBT adapted to eating disorders provides better control over one's eating and a more sophisticated way of treating body, figure and weight [2, 3].

### Study context

In the present study, a 10‐week pre‐designed group programme was offered to outpatients in a community mental health centre on the west coast of Norway [1]. The programme consisted of weekly meetings with elements from CBT [2], AC [21] and therapeutic writing [15]. In the therapeutic writing exercises, the participants were introduced to a specific feeling followed by 15 min of writing about it and subsequently a group discussion. Therapeutic writing exercises related to the documentation of feelings and food intake were explored. The group leaders guided the patients into dialogues about the current affect by means of therapeutic writing. The therapeutic writing sessions focused on processes for understanding one's own overeating episodes in the context of feelings. The therapy also involved written documentation of food intake as homework to provide important information about the eating problem [2].

### Aim and research question

The aim of the study was to investigate therapeutic writing as experienced by patients in the context of a BED group programme focusing on AC.

Research question: What are the patients' experiences of therapeutic writing focusing on AC in the context of a BED group programme?

## METHODS

The design was inductive with a qualitative explorative method [29]. The study approach was phenomenological, searching for meaning in the experiences [30] and hermeneutic, where meaning was interpreted [29].

### The authors' pre‐understanding

The first (E.O.) and last (K.R.) authors are experienced clinical therapists well acquainted with group therapy and group management. The first author was not involved in the recruitment process and had limited knowledge of the group programme and BED. She did not know any of the participants, with the exception of one whom she knew from previous therapy. The second author (V.U.) was familiar with the programme as a researcher who had been involved in previous sub‐studies [1, 31, 32] related to the main study. However, she had never met the participants nor had she been involved in the clinic. The last author (K.R.) knew the participants as she led the group therapy programme. She was not involved in recruiting the participants for the present study, nor did she conduct any of the interviews. The second and last authors are experienced researchers familiar with qualitative methods.

### Participants

*Inclusion criteria*: completed 10 weeks of the current group programme, >80% attendance, aged over 18 years, ability to speak and understand the Norwegian language and current or previous Body Mass Index over 35. (Table 1).

**TABLE 1:** Description of the participants

Sex	Age	Marital status	Education/work/activity	Bariatric surgery	Previous writing experience
6 women 2 men	24–62 years Average 42.5	Single (*n* = 5) Married (*n* = 3)	Employee (*n* = 4) Student (*n* = 2) Disability benefit (*n* **=** 2)	Y (*n* = 2) N (*n* = 6)	Previous experience of writing a diary (*n* = 2) Blogging (*n* = 1) No writing experience (*n* = 2) Writing in a student context (*n* = 1) Work‐related writing (*n* = 2)

A strategic sample was used [33] and 14 participants who had been offered the identical programme and fulfilled the inclusion criteria were identified. Information letters inviting the participants to take part in the study and a consent form were provided and all who were invited agreed to participate. They were each assigned a number to maintain anonymity, which was used throughout the analysis to indicate their statements in order to keep track of the meaning units. As we adhered to the principles of saturation, we stopped at eight participants, who were selected by one independent mercantile staff member: after completing the eight interviews we deemed that we had sufficient good and rich data from an adequate sample of participants who were experts on the phenomenon of interest [34].

The following open‐ended questions were formulated in an interview guide in order to focus on the relevant themes:

- What were your overall experiences of the current group programme involving therapeutic writing?
  - Please tell me about your experiences of participating in the therapeutic writing activities.
  - Please tell me about the writing process: what it was like to start writing, what it was like to write about your own feelings, and did you like elaborating on something from the activity?
  - Please describe the conditions that promoted and inhibited your writing. In what ways could the managers help you with your writing process?

The interviews were followed up by probing questions such as: can you please tell me a little more or give any examples of that?

The information letter guaranteed anonymity and stated that the participants could withdraw at any time without consequences for their future treatment and if necessary they would be offered a follow‐up conversation with their therapist. No follow‐ups were reported.

One pilot interview was performed and included in the study. The semi‐structured interview guide consisted of 13 questions and the interviews lasted from 24 to 57 min. The data were transcribed verbatim by the first author (E.O.) and discussed with the second (V.U.) and last (K.R.) authors.

### Analysis

Qualitative content analysis was used [35]. The interviews were conducted in 2019 and transcribed by the first author who also conducted the first steps of the analysis, that is coding and categorising the transcribed text, while bearing the research question in mind. Thereafter, the analysis process involved continuous interpretation and discussions between the three authors to achieve consensus in their attempt to find new ways of understanding the results. This process involved thematisation of the codes and reading the meaning units several times, after which the three authors searched for the latent meaning in the sub‐themes [35]. A main theme allows a movement in the abstraction process through the hermeneutical process [36]. An attempt was made to keep the final presentation of the findings close to the meaning described by the participants, while at the same time ensuring that the meaning did not disappear [37].

### Ethical considerations

The study was conducted in line with general ethical guidelines and the Declaration of Helsinki [38] and approved by the Regional Ethics Committee (REK no 2018/1773) and the Data Protection Authority at the hospital. The participants signed a written consent form, which stated that participation was voluntary, that they could withdraw at any time and that they were guaranteed confidentiality. The recorded interviews were stored in accordance with ethical standards and destroyed at the end of the study as set out in the regulations pertaining to identifiable personal information [29]. All data were anonymised and the participants were described on group level to ensure anonymity (Table 1). All participants are referred to as ‘he’ to prevent detection.

## FINDINGS

The following four findings describe how the participants involved themselves at a personal level in the writing process: *Struggling to achieve a flow in the writing process, Deeper understanding of eating patterns through writing, Moving specific feelings towards the surface by writing* and *Greater insight into oneself as a human being by shared writing*.

The main theme, *Therapeutic writing in a binge eating disorder programme means focusing on oneself as human being by becoming closer to one's feelings*, is based on an interpretation of the four sub‐themes.

An overview of the findings is presented in Table 2.

**TABLE 2:** Overview of the findings

Main theme	Therapeutic writing in a binge eating disorder programme means focusing on oneself as a human being by coming closer to one's feelings			
Sub‐themes	Struggling to achieve a flow in the writing process	Deeper understanding of eating patterns through writing	Moving the specific feeling towards the surface by writing	Expanding insight into oneself as a human being by shared writing
Categories	Expectations and instructions	Documentation of food intake is a necessary evil Disclosing avoidance strategies Clarifying matters	When unexpected feelings occur Confused by feelings Stronger awareness of affects	Input from others on own experiences Listening to and providing feedback on others' experiences

### Struggling to achieve a flow in the writing process

Some participants described how they struggled to express themselves, despite being familiar with writing in their daily life. Several had become emotionally overwhelmed while writing, which led to difficulties expressing themselves during the exercise.

I was incapable of writing because of the chaos I felt inside me. It was as if someone was pouring a bucket of something over me (7). It was "written in my head» almost as if I had managed to write, but could not express myself on paper because of being emotionally overwhelmed (7)

They had different expectations and experiences of writing and highlighted the importance of instructions, of being introduced to a topic and having enough time to write, as well as being free to write about their individual thoughts on the topic. The fact that the therapist presented feelings in a certain order was valued, as was having the therapist available during therapeutic writing.

I did not understand the meaning of writing, but suddenly you realise…The fact that someone is looking at you during the writing sessions made me write. It turned out that it had an effect and we got in‐depth information about the connections between food and feelings (8).

Those who were good at writing valued achieving a flow, writing themes in black and white and obtaining rich material.

I did not know what to write in advance, but it felt like writing in a flow… Things I had not really thought of suddenly found their place. One formulation led to something else, just like a good conversation (6). In retrospect, it was difficult to see how you ended up in a place you did not think you would end up in (1).

Writing by hand gave a more personal touch to the assignment and greater involvement in the writing process. Several described that something other than what they had expected suddenly appeared, leading them into places they did not think they would end up in.

### Deeper understanding of eating patterns through writing

Observing oneself by documenting one's own food intake in written form gave a complete and impartial overview of what they ate.

Previously, I had no thoughts about my overeating patterns… no reflection just something that happened where I mentally closed my eyes (3).

Several only irregularly documented the food they ate and reported a negative experience with it. Some admitted that they skipped noting overeating episodes. In these cases, they reflected on being dishonest about their own eating pattern, delayed or skipped documenting the food consumed or only did so on good days.

The confirmation that you have failed is not pleasant and not good reading. I'm losing heart (2). It requires quite a lot from the log keeper, because it is pointless if you start cheating when noting the food you eat and you are only fooling yourself… The note book looks you straight in the eye and you can see patterns (1).

Documentation of feelings in addition to food felt meaningful and helped them to become aware of the function of food.

I needed more time to feel comfortable because of the deep shame inside me (5). It became clear to me that when you had an overeating episode you used food to avoid being in touch with feelings… For me it was a wake‐up call that made me aware of my automatic, unconscious pattern. I was able to see the connections to why I want to injure myself with food (3).

The participants reflected and seemed to understand their own overeating pattern in a new way.

### Moving specific feelings towards the surface by writing

In various ways the writing process led all the participants towards their concealed feelings.

Feelings hit me hard when poking around and what was below the surface emerged. I was in therapy to poke feelings to the surface (3). A house can look completely normal from the outside, but if you just open the door it is on fire and burning on the inside. You just cannot see it on the outside. The smoke has not come out of the chimney yet and this is how therapeutic writing works, you open the door and look inside (3).

They reflected on the importance of getting in touch with various concealed feelings one by one. They all reflected on suppressed feelings that they never admitted having and then, in the writing process, noticed completely new experiences about them.

Feelings are important, but I've never looked at them individually by isolating them, which provided knowledge and the hope of being able to cope with feelings on a higher level (1). For me, emotional writing was when I discovered I was angry and sad. Overeating is related to the feelings that I am unable to get hold of. So by using therapeutic writing I became aware of them and found processing feelings easier. My feelings are more integrated now, but I still have to allow them and enable myself to get in touch with them (8).

Therapeutic writing gave the participants stronger affect awareness and knowledge of the emotional register. Unknown feelings about which they were unaware could hit very hard when activated in cases where something undiscovered appeared below the surface in relation to their inner emotional life.

### Expanding insight into oneself as a human being by shared writing

All the participants described an expanded insight into themselves through the shared writing process.

You are very narrow‐minded when you are only inside your own inner world. I felt that even though the pen was floating across the paper everything came from me. By sharing with others you got so much extra that filled some knowledge gaps (6).

Three participants highlighted the importance of group discussion after writing, where experiencing exploration and questions from the group led to a sense of relief.

In the group sessions there were often questions and exploration regarding topics. It almost felt like someone else was doing the thinking and seeing aspects that you yourself had not yet noticed. Through others, you became aware of something you might not otherwise have thought of (1).

Some struggled with a writing block and felt emotionally overwhelmed. In these cases, they highlighted the value and importance of group discussion after the therapeutic exercise.

I could relate to other group members' writing. They could write thoughts I had, but could not formulate on paper. I had to listen to what the others had written before I managed to find my own inner writing. (7).

They valued how they complemented each other as a group, increasing reflection through sharing with and listening to each other, better understanding themselves through such sharing. Input from others who might have experienced similar situations or were able to see things from an outside perspective was emphasised as valuable.

## DISCUSSION

The aim of this study was to investigate therapeutic writing focusing on AC as experienced by patients in the context of a BED group programme. Four sub‐themes describe the experience of writing, which is captured in the abstracted main theme; *Therapeutic writing in a binge eating disorder programme means focusing on oneself as a human being by becoming closer to feelings*. The overall variation in this experience describes a movement, both as a struggle and relief when seeing the words written in black and white and combining writing and dialogue in the group. Involving and focusing on oneself illuminates how the emotional process with oneself as human being is affected by writing about feelings. The four sub‐themes highlight the complexity of the relationship to oneself and one's own eating disorder. It seems as if focusing on feelings in therapeutic writing leads to one coming closer to oneself as a human being. In this sense, it means getting to know one's own feelings. This humanisation process seems to be a common thread in the findings.

The sub‐theme, *Struggling to achieve a flow in the writing process,* describes how expectations, structure and writing instructions affect the writing process. According to Furnes and Dysvik [13], concentrated writing sessions are important when implementing therapeutic writing. Seih, Chung and Pennebaker [39] highlight the value of writing about the same perspective repeatedly in order to create more emotions related to a given theme and ‘boost’ emotions for those who are easily disconnected from their feelings.

This sub‐theme also involves difficulties with writing. Writing can surpass the writer's cognitive abilities and low self‐esteem [40]. Our study indicates that processes associated with emotional difficulties and writing problems seem to be closely linked. These findings are in accordance with previous research. For instance, writers with a blockage report low levels of positive, constructive mental imagination and a lower level of liveliness in their ongoing mental performance [40].

According to Furnes and Dysvik [13], writing‐related disadvantages should be taken into account. Painful and difficult feelings and thoughts, as well as being unfamiliar with the written form, can be experienced as an additional burden, making it hard to start writing [13]. A way of overcoming writer's block is to be explicitly aware that this stage will pass and writing will become easier [41]. In our programme, different ways of solving writer's block were introduced: ‘inner writing’ consisting of scribbling without words on the paper, rewriting and reformulating words on the paper or only writing key words; holding the pen in constant movement, but in different ways. Our study indicates that the individuals can find their own way of coping with the writing problem. However, clinicians should be aware that writing may trigger emotional turbulence and formulation difficulties. Therapeutic writing can help, no matter how much or little one writes, and one unexpected finding is that the writing process was highly valued, even by those who reported that writing was difficult and strenuous. We interpret this finding as helpful on an unconscious level, regardless of whether or not one enjoys writing, which is supported by previous research concluding that although some do not like writing, it can be helpful for advancing the therapy process [42]. This is in accordance with Branch [43], who illustrates how the pen follows the mind of the writer in all directions and one can put anything on paper. If one trusts the writing hand the words can suddenly appear like a flash of light from nowhere [43].

The sub‐theme, *Deeper understanding of eating patterns through writing,* addresses experiences of understanding new mechanisms related to overeating and revealing self‐sabotage. Matz and Frankel [44] claim that tolerance for inconsistent affective states may mean that one no longer needs to resort to food to work through unacceptable feelings. One cannot control overeating without understanding and recognising the self‐regulatory function of overeating by experiencing feelings directly, where the capacity to regulate the intensity of feelings develops through the treatment process [44]. This sub‐theme reveals how understanding the mechanisms of overeating and self‐sabotage through therapeutic writing may be clarified through nuanced experiences of documenting one's food intake and such documentation seems to be an effective, concrete, helpful, but difficult writing exercise. Documenting one's food intake seems to be particularly important. The nuanced experiences of food documentation indicate that it reveals a complete and impartial overview of the actual food intake. It also provides an opportunity to recognise one's eating pattern, where there may be benefits related to being conscious of overeating episodes, even if it is tough. This is in line with Fairburn [2], who claims that by documenting all overeating episodes and honestly confronting the eating problem changes will emerge. If one does not document one's food intake, it is easy to pretend that one does not eat very much [45]. This study shows that getting into the habit of writing makes one aware of the triggers for overeating. This agrees with Borkin [42], who states that BED is based on underlying emotional reasons. Therapeutic writing offers several ways to grasp feelings where food documentation can give the desire for food a voice by creating a dialogue with feelings and recognising the role of food in sensing emotions [42].

Our study reveals that documenting food intake leads to an understanding of the eating pattern, thus therapeutic writing helps one to grasp what actually happens during overeating. The documentation of feelings enables one to become aware of an automatic eating pattern. When patients feel that overeating just happens, documenting food intake can function as awareness, mapping tools, retrieval options and anchoring. This highlights the importance of leading patients into greater AC, where therapeutic writing verbalises deep and hidden feelings. Our findings show that understanding of an eating pattern may occur during documentation of food intake. Wold and Uverud [46] point out that therapeutic writing forces the writer to become both physically and mentally aware of what is going on inside her/himself.

The sub‐theme *Moving the specific feeling towards the surface by writing* addresses experiences of disclosing the undiscovered through therapeutic writing, being led towards the unforeseen in new directions and feeling improved contact with emotional qualities. This sub‐theme indicates that when writing about feelings something other than that anticipated emerges or being influenced in ways that one was not prepared for. According to Bolton et al. [47] dynamics below the surface influence therapeutic writing, while the therapeutic processes referred to in this study, both therapeutic writing and AC, have mechanisms to drill into the deeper layers of the human being. Our study reveals how deep material emerges from below the surface, providing new information about more and different feelings of which one was previously unaware. Writing is described as helpful for grasping and validating feelings. This is in line with Kerner and Fitzpatrick [48], who emphasise that writing can encourage and modulate emotional intensity and create meaning and context, where the challenge is to tolerate difficult emotions and still continue writing [48]. By writing, emotions go through a real transformation where they are made visible [17]. The consistent affect focus in each group meeting is present in this study. By repetition, the affect focus is learned by patients so that they may relate to feelings in a more appropriate way without the therapist's presence [19].

Our study refers to the importance of isolating and getting in touch with each feeling on its own in order to get to know them better and be able to distinguish them from each other.

The sub‐theme *Expanding insight into oneself as a human being by shared writing* refers to the fact that one's writing is complemented by other group members, as the group increases and validates one's own reflections through sharing thoughts. This sub‐theme points to the importance of hearing other's experiences, recognition, discussion, sense of community and being affiliated. Writing can be a way to see feelings and experiences in perspective, where in groups one can experience a common understanding of the feelings [49]. In the present study the participants talked about their writing experiences in the group, but did not read their text aloud to the group. However, Bolton et al. [47] find that one can benefit from others by reading aloud what one has written in a group discussion after therapeutic writing.

Our programme recommends talking about the written text rather than reading it aloud as a script, in order to maintain the freedom to change the story along the way if necessary. Group members seem to bring out aspects and help one to grasp something one has not even thought about. Carefully facilitated writing and group work can make the group members perceive the previously unperceived that lies in front of their eyes without them seeing it. They may hear others articulating thoughts and feelings they have not completely formulated or identified themselves. These can provide opportunities for support, differentiation and expression of feelings, which reduces the need for overeating. Our study demonstrated that one can relate to the written work of others, experiencing that others can write thoughts that one recognises but failed to formulate. Therefore, writing is considered useful regardless of one's own ability to write.

### Methodological considerations

The material provided sufficient depth and information power to shed light on the research question in a complementary way [50]. A weakness in the sample may be the low gender representation of men and the fact that the programme was only tested locally. Mainly women applied for the group programme, so the disparity between men and women corresponds with applications for treatment. Two of the researchers had no direct relationship to the participants.

The method includes patients' reflections on treatment in a naturalistic setting and describes various ways in which the patients experience therapeutic writing [51]. A weakness is that we mainly highlight the positive experiences of therapeutic writing. The discussion is mostly related to previous studies on therapeutic writing, hence—a more specific discussion related to evidence‐based treatment and CBT for those with BED might have strengthened our study. User involvement is highlighted when patients are invited to take part in open‐ended reflections, as they have to decide what to highlight in the interviews. Qualitative methods study depth, which may not elicit hard facts. For this reason, our study cannot be considered representative, which is a limitation. However, small scale studies are important as a means of evaluating naturalistic setting [52] or bridging gaps between research and practice [53]. From this perspective, our study provides information about how to conceptualise therapeutic writing for patients suffering from BED as experienced in a group setting. Even though the results are not generalisable, they should be considered meaningful in the evaluation of BED intervention strategies [53]. The study could be strengthened by using several focus groups or individual interviews, investigating the same intervention from other perspectives and with participants who took part in the same programme.

## CONCLUSION

This study concludes that as a result of therapeutic writing the participants were engaged in focusing on themselves as a human being and had nuanced experiences of being aware of feelings below the surface. The findings show that therapeutic writing in treatment of BED is valuable and can help the persons to come to terms with thoughts, feelings and their eating pattern. A person's experiences of the complexity of the relationship to oneself and one's own eating disorder can develop into a humanisation process by therapeutic writing. It seems as if focusing on feelings in therapeutic writing has a potential to provide relief by making one aware of one's own feelings. The study clarifies that the combination of writing and conversation in a group discussion is of particular value and experienced as positive. The results underline the importance of participating in a group programme. Although patients find therapeutic writing demanding, difficult and strenuous, they can experience new ways of relating to their own feelings from a programme using writing exercises. This programme can easily be transferred to an international context and other health areas as we demonstrate that the use of writing exercises involves little effort and few disadvantages. We also show that the programme is both feasible and user‐friendly, has an ethical approach and can be transferred to a group whose members find it difficult to explore their own feelings in greater depth.

## IMPLICATIONS FOR PRACTICE AND FURTHER RESEARCH

This study indicates that AC can be transferred to other diagnostic groups and treatments. In particular, therapeutic writing about feelings may be transferable to other mental health settings. However, it is necessary to investigate the actual benefits of therapeutic writing in a quantitative intervention study and discuss its relevance in light of the international literature on BED.

New qualitative studies on therapeutic writing in the context of BED can provide a greater understanding of therapeutic writing as a tool. Research on therapeutic writing and AC combining quantitative and qualitative methods would be beneficial, in line with therapeutic writing as central in BED. Staff and patients in the field of group therapy, eating disorders and BED may be interested in how therapeutic writing can be implemented as a supplement in treatment programmes.

## AUTHOR CONTRIBUTIONS

EO was responsible for data collection and the first steps of analysis. EO and KR was responsible for writing the manuscript. VU provided feedback on the analysis, the draft of the manuscript and contributed to the content. All authors approved the final version and adhered to the criteria pertaining to the research process as recommended by the International Committee of Medical Journal Editors (http://www.icmje.org/recommendations).

## CONFLICT OF INTEREST

The author(s) declared no potential conflicts of interest with respect to the research, authorship and/or publication of this article.

## Data Availability

The findings from this study are supported by quotations selected by EO. More research data that support the findings from this study are not shared or publicly available due to ethical restrictions.
